# Supplementary material for: Potassium Ions Decrease Mitochondrial Matrix pH: Implications for ATP Production and Reactive Oxygen Species Generation
Source: Int J Mol Sci. 2024 Jan 19;25(2):1233. doi: 10.3390/ijms25021233 (PMC10815940; doi:10.3390/ijms25021233)
Supplement: Supplementary file 1 [file ijms-25-01233-s001.zip › ijms-2780630-supplementary.pdf]

Figure S1

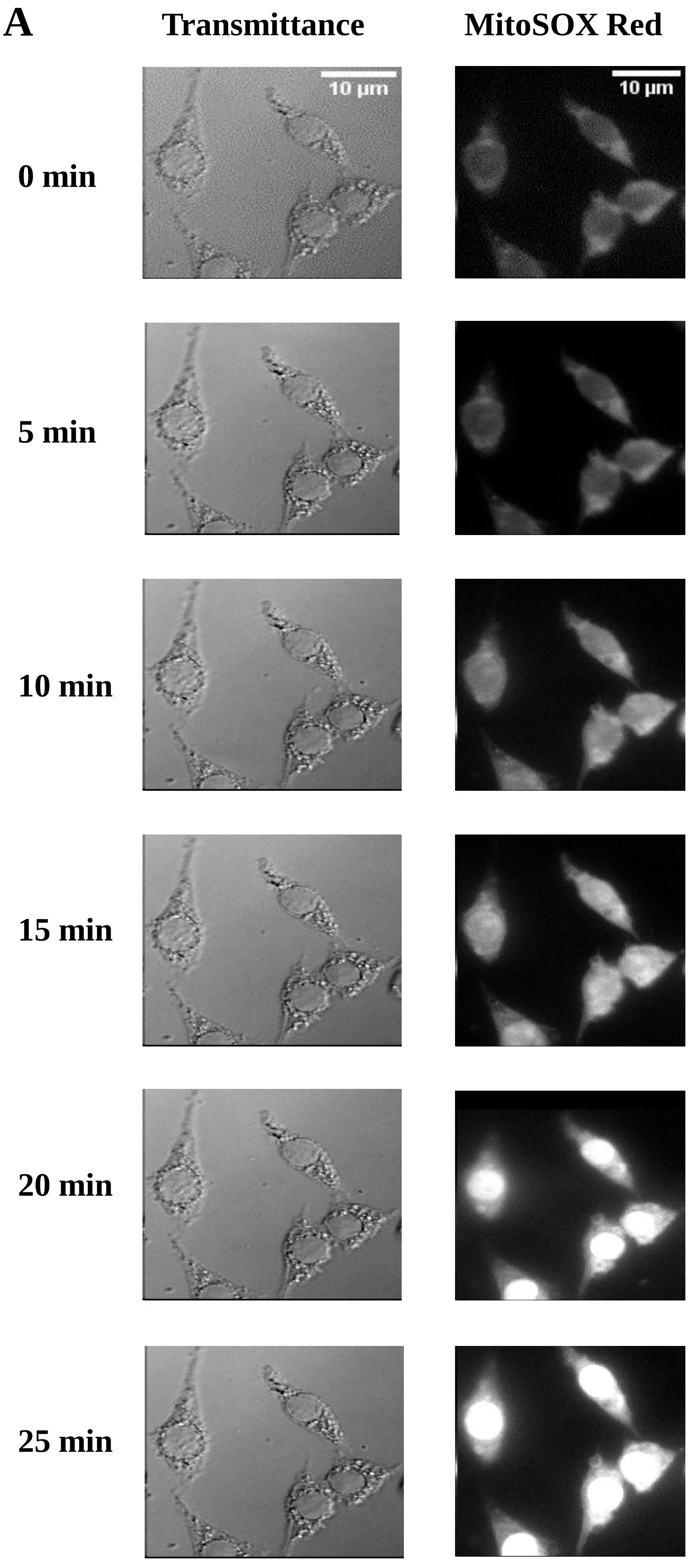

A
Transmittance
MitoSOX Red
10 µm
10 µm
0 min
5 min
10 min
15 min
20 min
25 min

Figure S1

B

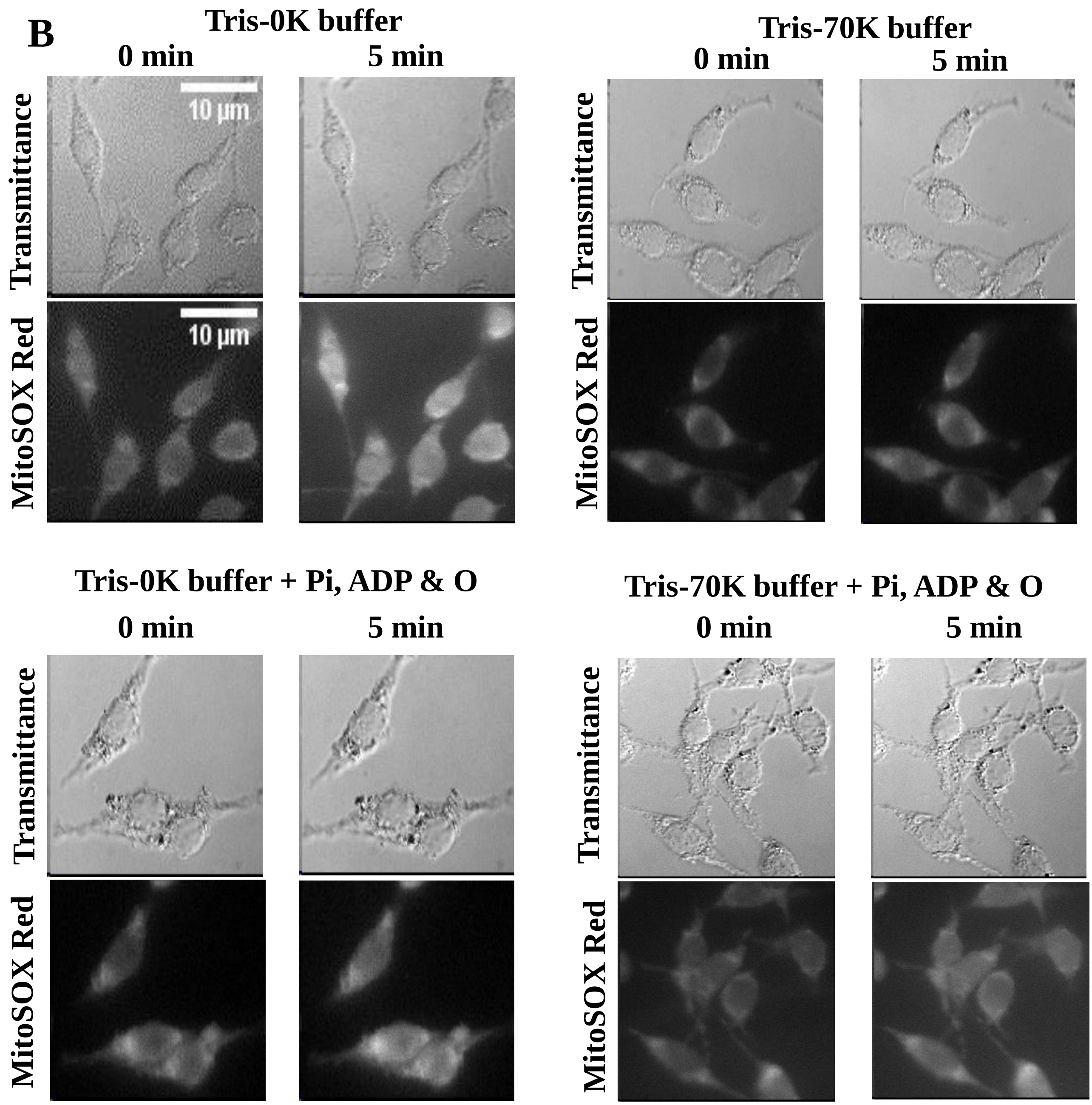
Tris-0K buffer
0 min
5 min
Tris-70K buffer
0 min
5 min
Transmitance
MitoSOX Red
10 µm
Tris-0K buffer + Pi, ADP & O
Tris-70K buffer + Pi, ADP & O

C

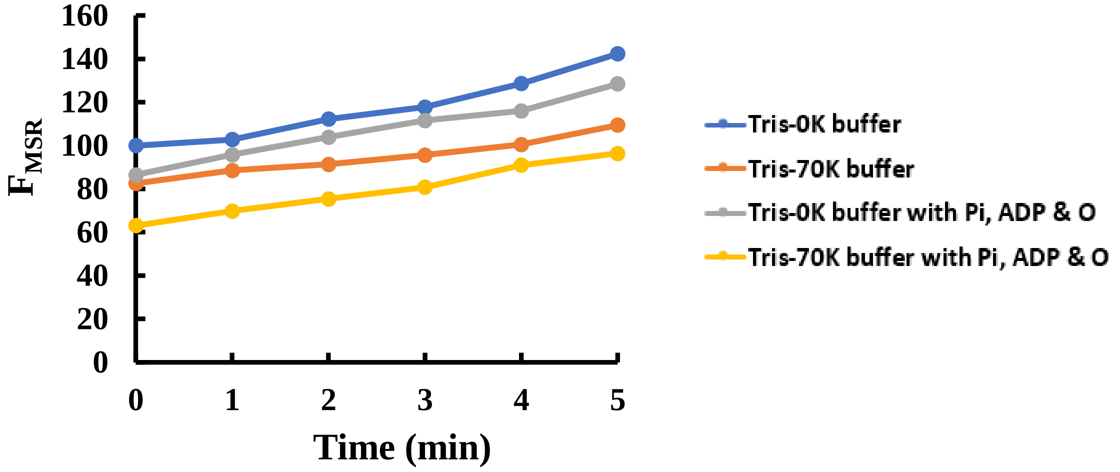
F_{MSR}
160
140
120
100
80
60
40
20
0
0
1
2
3
4
5
Time (min)
Tris-0K buffer
Tris-70K buffer
Tris-0K buffer with Pi, ADP & O
Tris-70K buffer with Pi, ADP & O

Figure S1

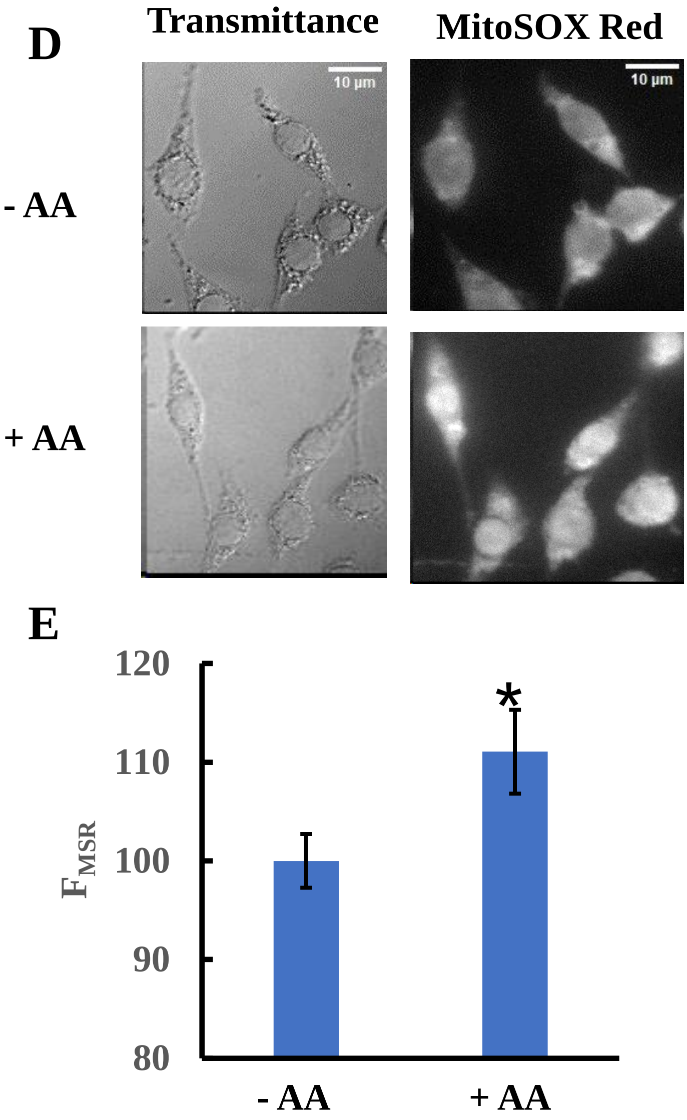

Figure S1. MitoSOX Red fluorescence in plasma membrane permeabilized cells. Prior to observation, permeabilized cells were incubated with 2.5 μM MitoSOX Red for 10 minutes at 25° C. The time indicated is the time since the start of observation. **A)** Time-dependent changes in fluorescence images of MitoSOX Red in cells incubated in Tris-0K buffer. The fluorescence intensity in mitochondria increased until t=10 min, but after 15 min the nuclei were stained. Therefore, fluorescence intensity was quantified at 5 or 10 min after the start of the observation. Bar, 10 μm. **B)** Comparison of MitoSOX Red fluorescence images between cells incubated in four different buffers. Fluorescence images were obtained at t=0 and 5 min. Pi (1 mM KH_2PO_4), ADP (0.5 mM KH_2ADP), and O (1 μM oligomycin). Bar, 10 μm. **C)** Time-dependent changes in F_{MSR} (the integrated fluorescence intensity of MitoSOX Red in single cells) measured in the four different buffers. The buffers used are the same as in Fig. S2B. The fluorescence value in Tris-0K buffer at t = 0 min was normalized to 100. **D)** Fluorescence images of MitoSOX Red in cells incubated with and without 1μM antimycin A. AA, antimycin A. Antimycin A was added to the cells incubated in Tris-0K buffer at t = 5 min, and the fluorescence intensity was measured at t=10min. Bar, 10 μm. **E)** MitoSOX Red fluorescence intensity. Intensity was measured in Tris-0K buffer. The fluorescence value in Tris-0K buffer without antimycin A was normalized to 100. The number of cells analyzed are 4 for without antimycin A and 5 for with antimycin A. Values represent the mean ± SEM. * $p < 0.05$ vs. without antimycin A.

Figure S2

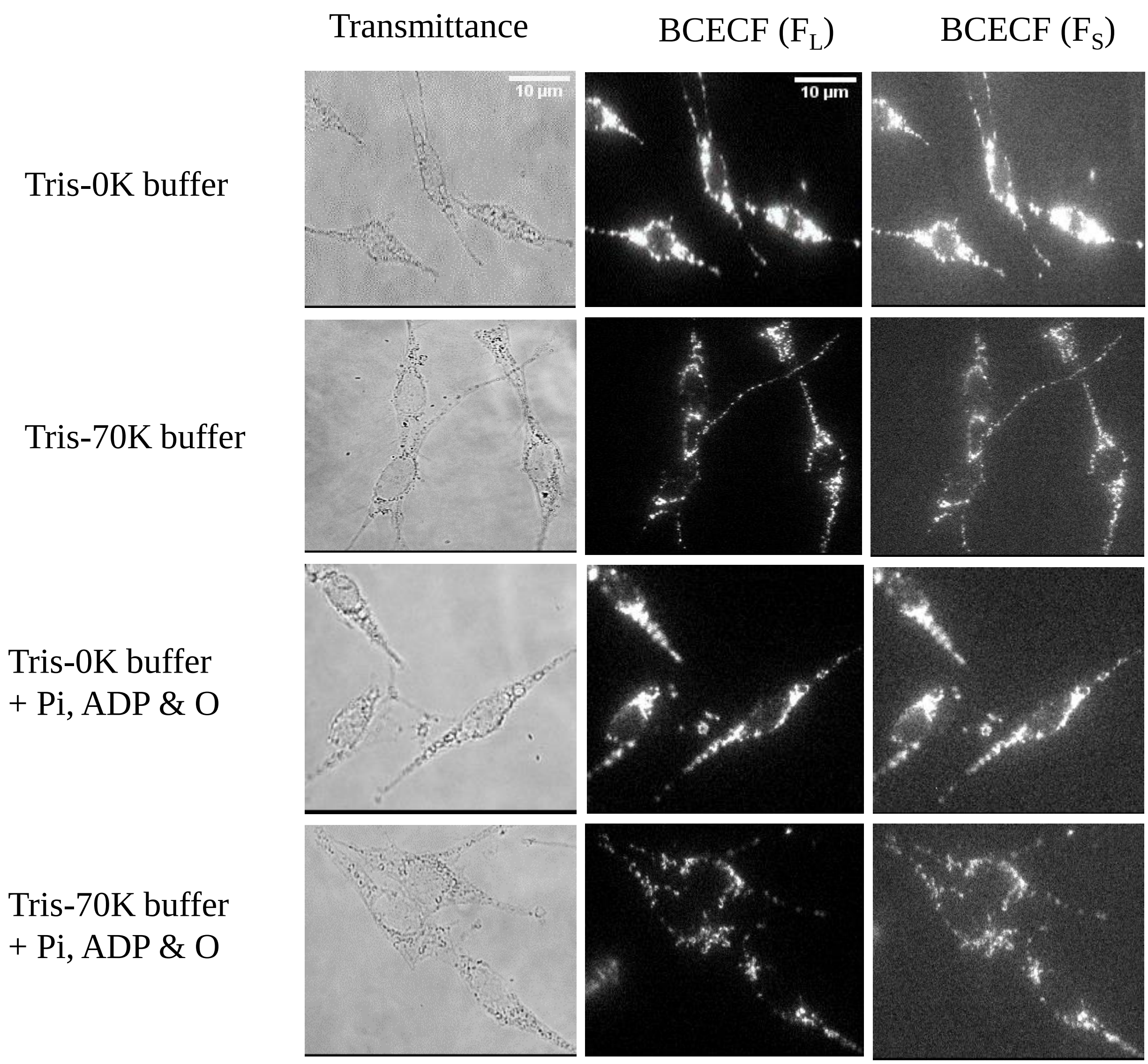

Figure S2. Fluorescence images of BCECF in plasma membrane permeabilized cells Fluorescence images of BCECF in the cells incubated in four different buffers. The buffers indicated are the same as those shown in Fig. S1B. F_L and F_S are defined in section 4.4. To show the clear distribution of BCECF fluorescence, the exposure time for BCECF images (Fs) is extended to 10 seconds. Bar, 10 μm.

Figure S3

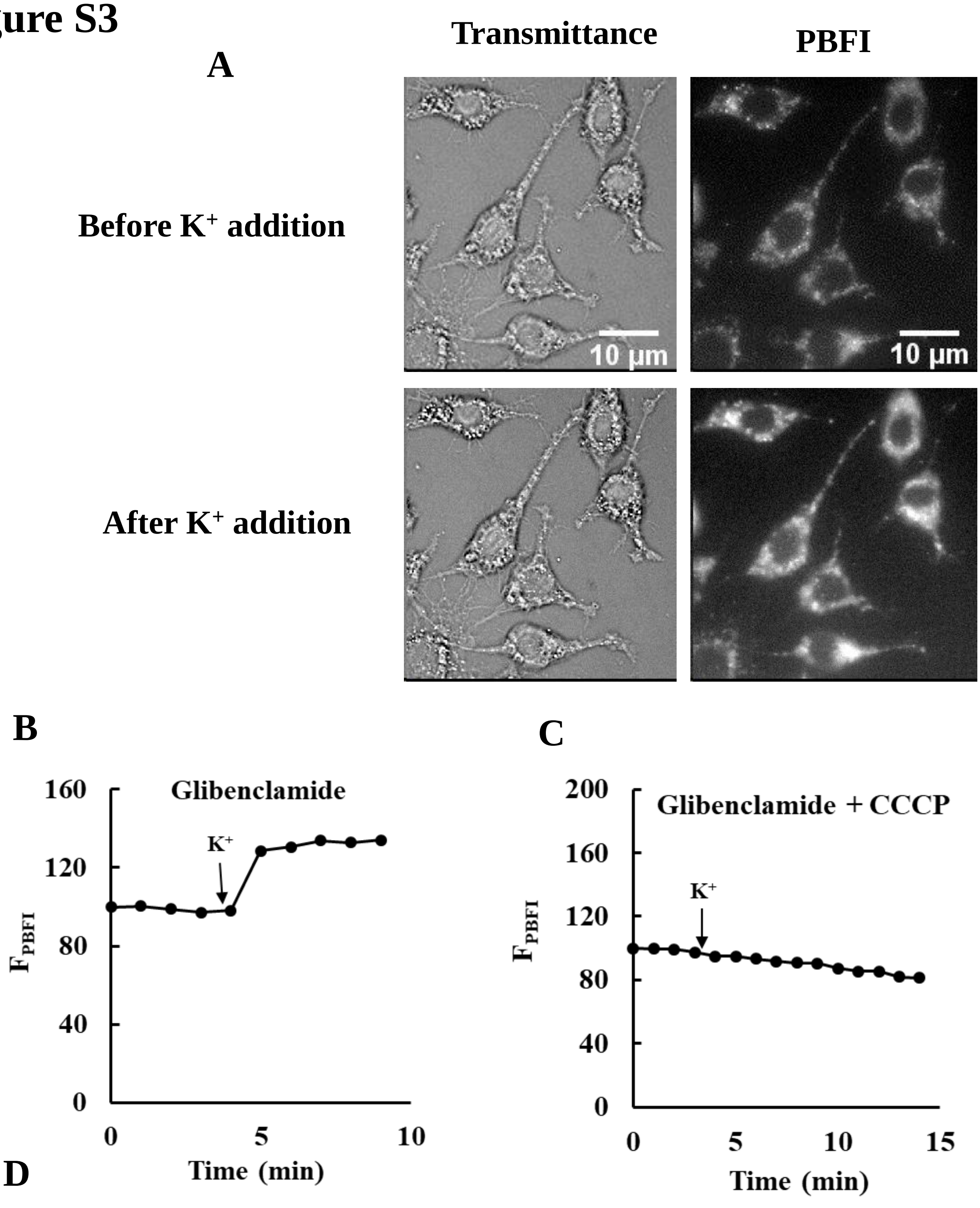

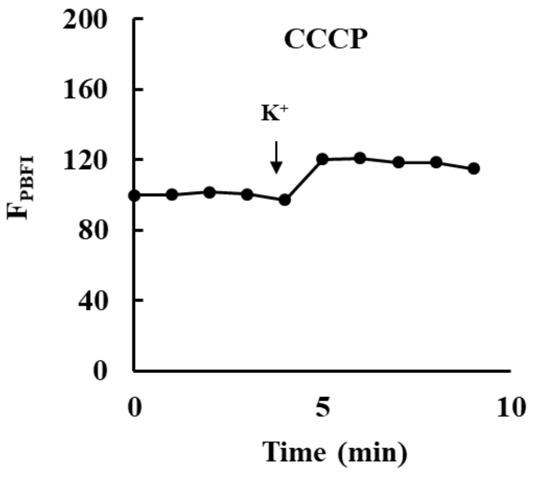

Figure S3. Changes in PBFI fluorescence in plasma membrane permeabilized cells.
A) Fluorescence images of PBFI before and after the addition of K^+. Bar, 10 µm. **B-D)** Effects of glibenclamide (B)**,** glibenclamide+CCCP (C) and CCCP (D) on the increase of PBFI fluorescence in mitochondria upon addition of 70 mM K^+. Arrows show the addition of K^+.

Figure S4

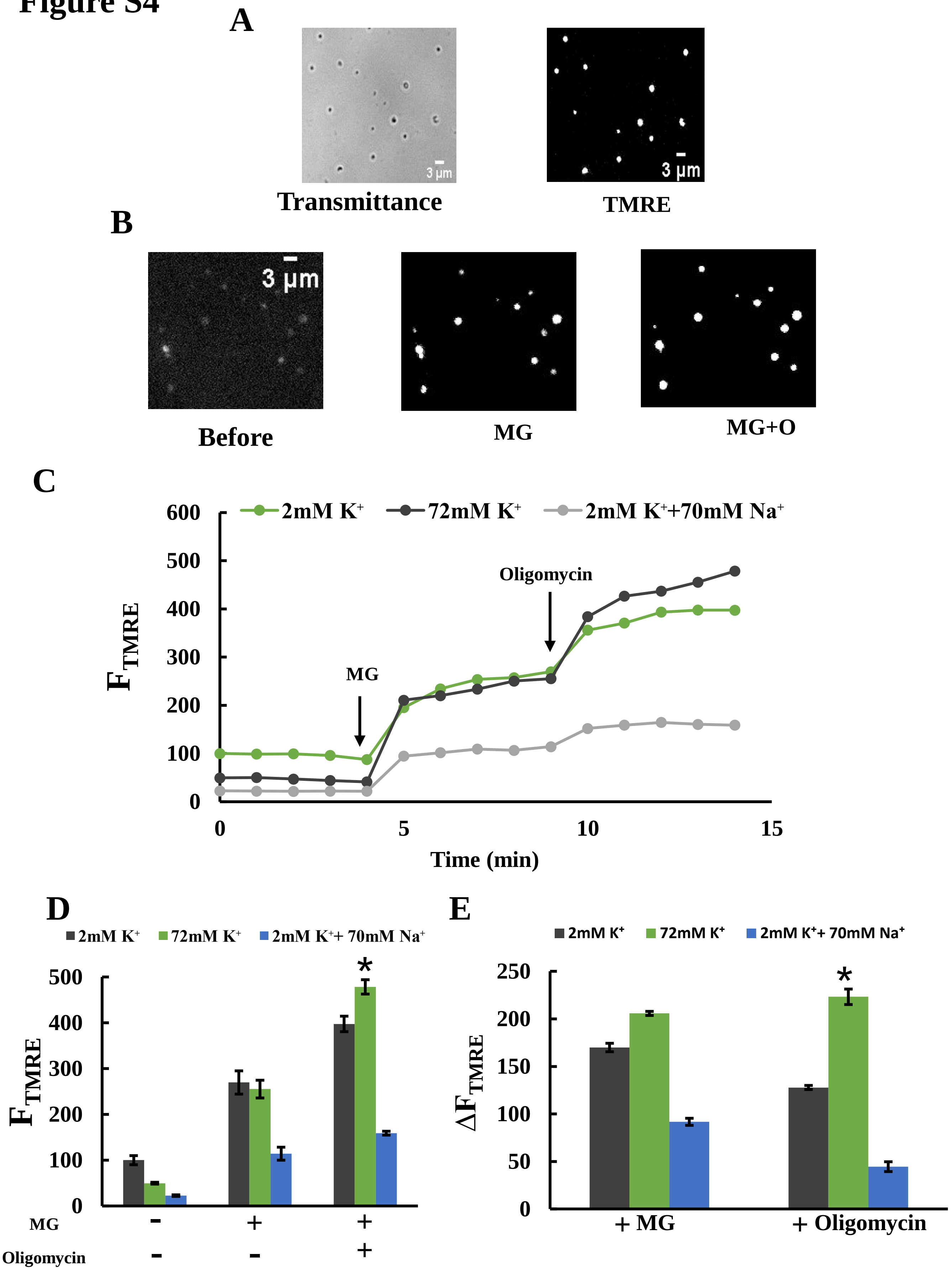
A
3 µm
3 µm
Transmittance
TMRE
B
3 µm
Before
MG
MG+O
C
2mM K+
72mM K+
2mM K++70mM Na+
F TMRE
Oligomycin
MG
0
100
200
300
400
500
600
0
5
10
15
Time (min)
D
2mM K+
72mM K+
2mM K++ 70mM Na+
F TMRE
0
100
200
300
400
500
*
MG
Oligomycin
-
+
+
-
-
+
E
2mM K+
72mM K+
2mM K++ 70mM Na+
ΔF TMRE
0
50
100
150
200
250
*
+ MG
+ Oligomycin

Figure S4. Effects of K^+ on $\Delta\psi_m$ of isolated mitochondria

(A) Optical images of isolated C6 mitochondria adsorbed on a glass bottom dish at the same microscopic field. Bar, 3 μm. **(B)** Changes in TMRE fluorescence images in a Tris-70K buffer with 1 mM KH_2PO_4, 0.5 mM KH_2ADP, and 1 mg/mL BSA. TMRE fluorescence images were obtained before and after adding 5 mM malate and 5 mM glutamate (MG) and after further adding 1 μM oligomycin (MG + O). Bar, 3 μm. **(C)** Time-resolved TMRE fluorescence changes in isolated mitochondria. MG and oligomycin were added at t = 4 and 9 min, respectively. TMRE fluorescence was measured in 3 different buffer conditions, as explained in Fig. 4A. Each buffer contains 1 mM KH_2PO_4 and 0.5 mM KH_2ADP. The average value of F_{TMRE} in a 2 mM K^+ buffer before adding malate and glutamate (MG) was set to 100. **(D, E)** TMRE fluorescence (D) and TMRE fluorescence changes (E) upon adding MG and oligomycin in isolated mitochondria. Three independent experiments were performed for each condition, with >50 mitochondria analyzed in each experiment. Values represent the mean ± SEM (n = 3). * $p < 0.05$ vs. 2mM K^+ buffer for respective conditions.

Figure S5

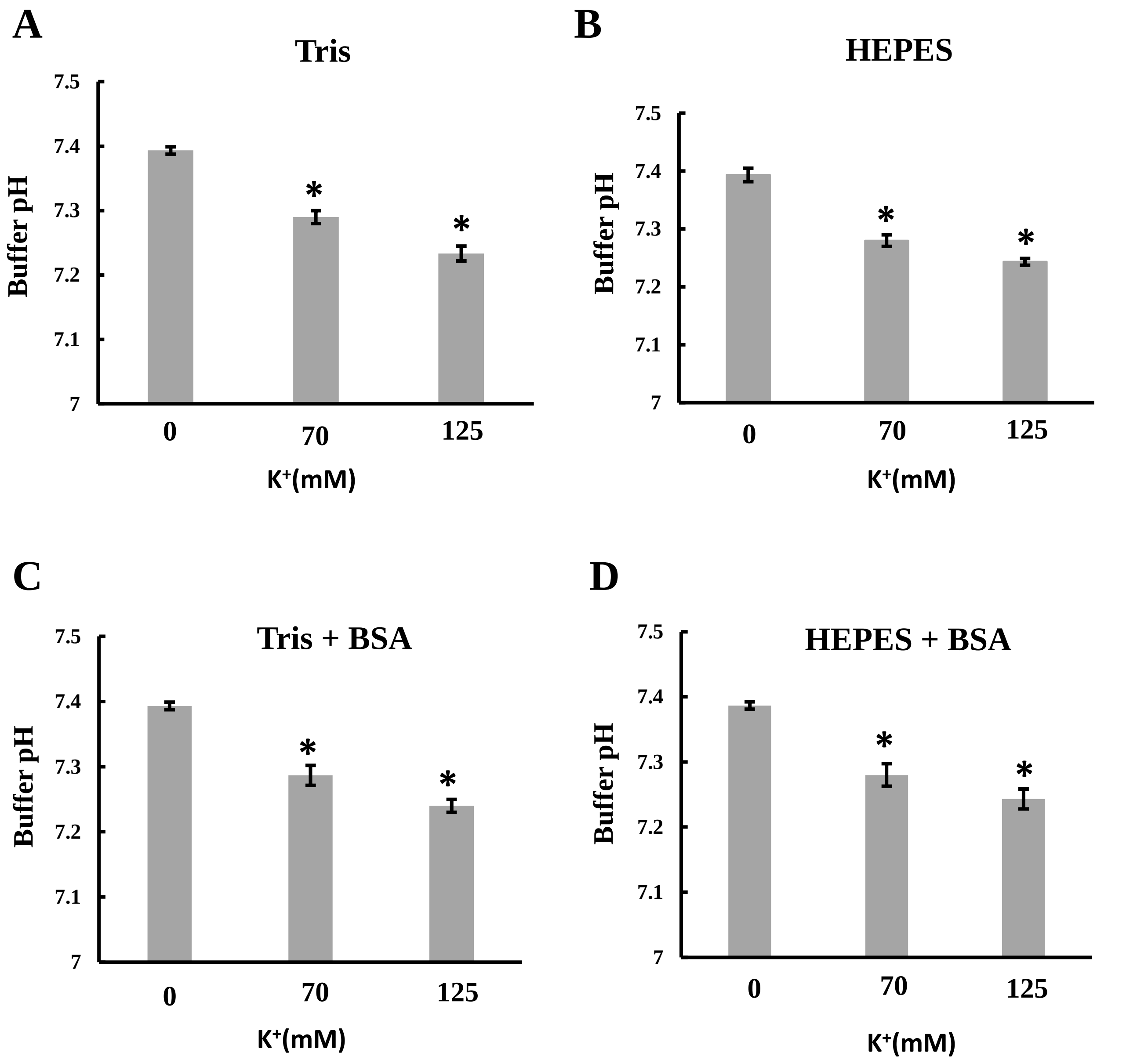

Figure S5. Effects of K^+ on the pH of buffer solution

The pH of the solution was measured as described in Fig. 7. **(A)** 1 mM Tris-HCl, **(B)** 1 mM HEPES-KOH, **(C)** 1 mM Tris-HCl with 10 mg/mL BSA, **(D)** 1 mM HEPES-KOH with 10 mg/mL BSA. Values represent the mean ± SEM (n > 3). * $p < 0.05$ vs. 0mM K^+ for **(A,B,C,D)**.

Figure S6

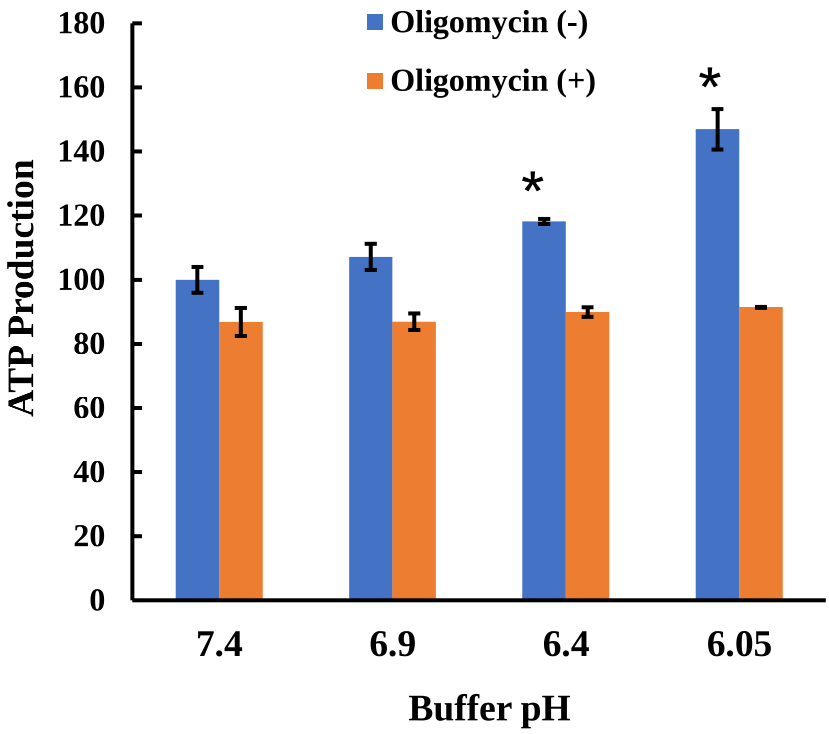

Figure S6. Effects of buffer pH on ATP production

ATP production was measured in isolated mitochondria in a buffer (pH 7.4, 6.9, 6.4 and 6.05 with or without oligomycin. Values represent the mean ± SEM (n > 3). * $p < 0.05$, vs. pH 7.4 for the respective conditions.

Figure S7

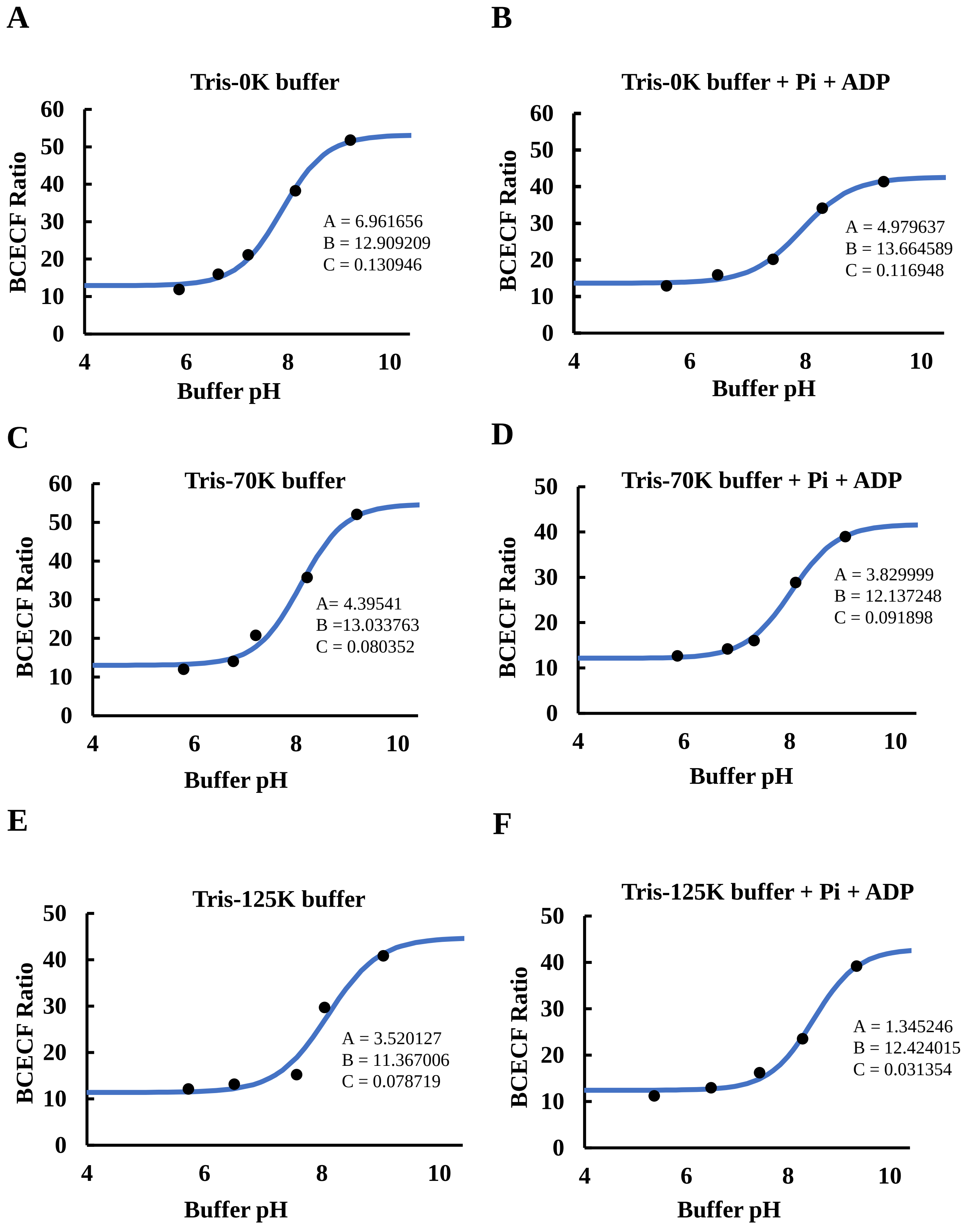
A
Tris-0K buffer
BCECF Ratio
0
10
20
30
40
50
60
4
6
8
10
Buffer pH
A = 6.961656
B = 12.909209
C = 0.130946
B
Tris-0K buffer + Pi + ADP
BCECF Ratio
0
10
20
30
40
50
60
4
6
8
10
Buffer pH
A = 4.979637
B = 13.664589
C = 0.116948
C
Tris-70K buffer
BCECF Ratio
0
10
20
30
40
50
60
4
6
8
10
Buffer pH
A= 4.39541
B =13.033763
C = 0.080352
D
Tris-70K buffer + Pi + ADP
BCECF Ratio
0
10
20
30
40
50
4
6
8
10
Buffer pH
A = 3.829999
B = 12.137248
C = 0.091898
E
Tris-125K buffer
BCECF Ratio
0
10
20
30
40
50
4
6
8
10
Buffer pH
A = 3.520127
B = 11.367006
C = 0.078719
F
Tris-125K buffer + Pi + ADP
BCECF Ratio
0
10
20
30
40
50
4
6
8
10
Buffer pH
A = 1.345246
B = 12.424015
C = 0.031354

Figure S7. Calibration curve for determining matrix pH from BCECF fluorescence ratio

F_S/F_L ratios of BCECF in permeabilized cells were measured in buffers with different pH in the presence of 5 μM CCCP without adding respiratory substrates. The buffers used were (A) Tris-0K buffer, (B) Tris-0K buffer + Pi (1 mM KH2PO4) + ADP (0.5 mM KH2ADP), (C) Tris-70K buffer, (D) Tris-70K buffer + Pi + ADP, (E) Tris-125K buffer, (F) in Tris-125K buffer + Pi + ADP. The dots represent the experimental data. The solid line shows the theoretical curve of $(F_L / F_S) = (A + B \times 10^{(7\text{-pH})}) / (C + 10^{(7\text{-pH})})$, which was fitted to the experimental data using least-squares fitting.
